# Air pollution exposure during preconception and first trimester of pregnancy and gestational diabetes mellitus in a large pregnancy cohort, Hebei Province, China

**DOI:** 10.3389/fendo.2024.1343172

**Published:** 2024-09-11

**Authors:** Mei-Ling Tian, Ying Jin, Li-Yan Du, Gui-Yun Zhou, Cui Zhang, Guo-Juan Ma, Yin Shi

**Affiliations:** ^1^ Department of Obstetrics and Gynecology, Hebei General Hospital, Shijiazhuang, China; ^2^ Department of Information Management, Hebei Center for Women and Children’s Health, Shijiazhuang, China; ^3^ Department of Obstetrics and Gynecology, Hebei Provincial Hospital of Chinese Medicine, Shijiazhuang, China

**Keywords:** air pollution, gestational diabetes mellitus, PM2.5, PM10, Hebei

## Abstract

**Objective:**

To explore the relationship between the exposure level of particulate matter 2.5 (PM2.5) and particulate matter 10 (PM10) in the air of pregnant women during preconception and first trimester of pregnancy and the risk of gestational diabetes mellitus (GDM).

**Methods:**

The data of pregnant women delivered in 22 monitoring hospitals in Hebei Province from 2019 to 2021 were collected, and the daily air quality data of their cities were used to calculate the exposure levels of PM2.5 and PM10 in different pregnancy stages, and logistic regression model was used to analyze the impact of exposure levels of PM2.5 and PM10 on GDM during preconception and first trimester of pregnancy.

**Results:**

108,429 singleton live deliveries were included in the study, of which 12,967 (12.0%) women had a GDM diagnosis. The prevalence of GDM increased over the course of the study from 10.2% (2019) to 14.9% (2021). From 2019 to 2021, the average exposure of PM2.5 and PM10 was relatively 56.67 and 103.08μg/m3 during the period of preconception and first trimester of pregnancy in Hebei Province. Handan, Shijiazhuang, and Xingtai regions had the most severe exposure to PM2.5 and PM10, while Zhangjiakou, Chengde, and Qinhuangdao had significantly lower exposure levels than other regions. The GDM group had statistically higher exposure concentrations of PM2.5 and PM10 during the period of preconception, first trimester, preconception and first trimester (P<0.05). Multivariate logistic regression analysis showed that the risk of GDM increases by 4.5%, 6.0%, and 10.6% for every 10ug/m3 increase in the average exposure value of PM2.5 in preconception, first trimester, preconception and first trimester, and 1.7%, 2.1%, and 3.9% for PM10. Moreover, High exposure to PM2.5 in the first, second, and third months of preconception and first trimester is associated with the risk of GDM. And high exposure to PM10 in the first, second, and third months of first trimester and the first, and third months of preconception is associated with the risk of GDM.

**Conclusion:**

Exposure to high concentrations of PM2.5 and PM10 during preconception and first trimester of pregnancy can significantly increase the risk of GDM. It is important to take precautions to prevent exposure to pollutants, reduce the risk of GDM, and improve maternal and fetal outcomes.

## Introduction

Gestational diabetes mellitus (GDM) is one of the most common pregnancy complications and usually diagnosed in the second and third trimester. It was defined as carbohydrate intolerance of any degree with onset or first recognition during pregnancy (1). GDM increases not only the rate of adverse maternal and infant outcomes, but also the long-term risk of childhood obesity, type 2 diabetes and cardiovascular disease (2). which has a serious impact on both mothers and fetuses. With the adjustment of fertility policy, the number of pregnant women with advanced maternal age and pregnancy complications is increasing, and the incidence rate of GDM is increasing. The GDM incidence in China was reported to be 11.91% (3). How to prevent GDM is a serious public health challenge.

Air pollution is one of the most serious environmental problems in the world. Research shows that atmospheric particulate matters are one of the main risk factors for diabetes (4, 5). Although the pathophysiological mechanisms between ambient air pollution and maternal disease remain unclear, several mechanisms including systemic inflammation, oxidative stress, and endothelial dysfunction have been proposed (6, 7). Systemic inflammation and oxidative stress induced by air pollution can lead to insulin resistance, which is the underlying mechanism of GDM (8).

The susceptibility of pregnant women to pollutants increases due to physiological characteristics such as increased blood volume and accelerated respiratory rate during pregnancy. This study analyzes the data of pregnant women in 108, 429 singleton live deliveries in Hebei Province, and expounds the relationship between air pollution exposure of women during the period of pre-pregnancy and first trimester and the occurrence of GDM. The aim is to provide scientific basis for preventing exposure to pollutants, reducing the risk of GDM, improving maternal and fetal outcomes, and improving the quality of the birth population.

## Methods

### Study area

Hebei Province (36°05′–42°40′N, 113°27′–119°50′E), which encompasses the areas of Beijing and Tianjin, has the Bohai Sea to the east, Yanshan Mountains to the north, and Tai hang Mountains to the west.

### Data collection

This is a retrospective study. Data in this study were retrieved from the monitoring information management system for pregnant women in 22 hospitals of Hebei Province China from 2019 to 2021. And the 22 monitoring hospitals are distributed in 11 cities in Hebei Province.

Inclusion criteria are single live birth and over 28 weeks of gestation. Exclusion criteria included age <20y, stillbirth, multiple births, and incomplete data. 108,429 singleton live deliveries were included in the study.

The air pollutant concentration data (PM2.5, PM10) were obtained from China Environmental Monitoring Network (http://www.cnemc.cn/). The concentration was recorded hourly for each of the following air pollutants: particulate matters (PM) with a diameter of 10 µm or less (PM10, µg/m3), PM with a diameter of 2.5 µm or less (PM2.5, µg/m3). The study was approved by the ethics committee of Hebei Women and Children’s Health Center.

### Diagnostic approaches and criteria

Pregnant women at 24–28 weeks of gestation were tested for fasting 75-g oral glucose tolerance. GDM was diagnosed if one or more thresholds are met or exceeded: fasting blood glucose: 5.1 mmol/L, blood glucose of 1 hour: 10.0 mmol/L) and blood glucose of 2 hour: 8.5 mmol/L (9).

We calculated individual average concentrations of air pollutants in the following windows based on the gestational age of each pregnant woman::(1) preconception (12weeks before pregnancy, Pre-T); (2) first trimester (1–13 gestational weeks, T1).

### Statistical analyses

SPSS 21.0 software was used for statistical analyses. The data description was presented as median [interquartile ranges (IQR)] for continuous variables and rank sum test is used for the comparison between groups. χ^2^-test is used for the comparison between groups of counting data. The multivariate logistic regression model was used to analyze the risk factors of GDM. p was set at <0.05 for statistical significance.

## Results

### Maternal characteristics

In this study, 108,429 singleton live deliveries were included in the study, of which 12,967 (12.0%) women had a GDM diagnosis. The prevalence of GDM increased over the course of the study from 10.2% (4,751/46,453) in 2019 to 14.9% (4,129/27,661) in 2021. Women with GDM were more likely to be with advanced age, multigravidity, and PE (**Table 1**).

**Table 1:** Demographic characteristics by GDM for singleton deliveries from 2019 to 2021.

Characteristic	GDM	Non-GDM	χ 2	P
Maternal age			518.000	<0.001
13-19	18 (2.7%)	638 (97.3%)		
20-34	9987 (11.1%)	80197 (88.9%)		
35-55	2962 (16.8%)	14627 (83.2%)		
Marital status			0.914	0.339
Married	12907 (12.0%)	95075 (88.0%)		
Unmarried	60 (13.4%)	387 (86.6%)		
Gravidity			46.734	<0.001
0	3605 (10.9%)	29349 (89.1%)		
≥1	9362 (12.4%)	66113 (87.6%)		
Parity			1.539	0.215
0	5171 (12.1%)	37527 (87.9%)		
≥1	7796 (11.9%)	57935 (88.1%)		
PE			211.482	<0.001
No	12311 (11.7%)	92853 (88.3%)		
Yes	656 (20.1%)	2609 (79.9%)		
Anaemia			0.004	0.951
No	7621 (12.0%)	56132 (88.0%)		
Yes	5346 (12.0%)	39330 (88.0%)		

### Exposure of PM2.5&PM10 during the period of stage 1 and 2 among various cities in Hebei Province

From 2019 to 2021, the average exposure of PM2.5 was 56.67μg/m3 during the period of stage 1 and 2 in Hebei Province, the median was 58.83μg/m3, max 95.00μg/m3, with a minimum value of 18.83μg/m3. The average exposure of PM10 was 103.08μg/m3 during the period of pre-pregnancy and first trimester in Hebei Province, the median was 105.00μg/m3, max 165.67μg/m3, with a minimum value of 43.83μg/m3. The values of PM2.5 and PM10 are much higher than the WHO health standard (annual average concentration of 10μg/m3). From the perspective of regional distribution, Handan, Shijiazhuang, and Xingtai regions had the most severe exposure to PM2.5 and PM10, while Zhangjiakou, Chengde, and Qinhuangdao had significantly lower exposure levels than other regions (**Table 2**).

**Table 2:** Total exposure of PM2.5 and PM10 during the period of stage 1 and 2 in different cities in Hebei Province.

	PM2.5				PM10			
	Mean	Median	M(P25, P75)	Min ∼ Max	Mean	Median	M(P25, P75)	Min ∼ Max
Total	56.67 ± 13.63	58.83	58.83, 66.17	18.83, 95.00	103.08 ± 22.12	105.00	88.00, 119.00	43.83, 165.67
Baoding	58.31 ± 12.17	57.33	50.50, 72.17	31.33∼88.00	101.00 ± 16.48	99.00	88.50, 118.50	63.17∼141.33
Chengde	31.55 ± 4.17	31.33	29.33, 33.40	21.50∼44.33	67.08 ± 9.51	68.83	58.67, 75.83	48.17∼95.33
Shijiazhuang	61.74 ± 9,13	60.83	57.17, 68.17	39.17∼95.00	114.40 ± 14.43	115.50	106.00, 122.33	75.50∼165.67
Tangshan	55.77 ± 6.32	55.67	51.50, 59.67	39.50∼75.00	102.52 ± 9.28	103.67	96.83, 109.17	78.17∼129.33
Xingtai	61.51 ± 11.65	60.17	53.17, 68.83	35.50∼89.67	113.21 ± 19.30	112.33	97.00, 129.67	71.33∼160.17
Qinhuangdao	38.41 ± 4.52	39.00	36.00, 41.50	26.83∼48.67	71.08 ± 8.09	71.00	65.17, 77.67	53.50∼88.00
Zhangjiakou	26.25 ± 2.78	26.67	23.83, 28.17	18.83∼36.50	58.80 ± 8.61	57.00	53.33, 63.33	43.83∼87.50
Cangzhou	51.73 ± 7.75	50.00	46.83, 57.83	34.50∼68.50	90.97 ± 11.03	91.17	83.50, 101.00	60.83∼114.67
Hengshui	55.92 ± 8.37	54.33	51.83, 63.00	36.00∼77.67	92.72 ± 11.83	91.17	83.67, 100.17	60.50∼131.17
Handan	62.99 ± 10.63	61.33	56.17, 69.00	39.00∼88.00	109.65 ± 24.38	106.67	88.50, 128.50	59.83∼164.33

### Exposure of PM2.5 and PM10 in different groups

Compared to the non-GDM group, the GDM group had statistically higher exposure concentrations of PM2.5 and PM10 during the period of stage 1, stage 2, stage 1 & 2 (**Tables 3**, **4**), and per month (**Tables 5**, **6**) (P<0.05).

**Table 3:** Exposure of PM2.5 in different groups of pre-trimester, first-trimester, and pre-trimester&first-trimester.

	PM2.5				Z value	P
	GDM		Non-GDM			
	M(P25, P75)	Min ∼ Max	M(P25, P75)	Min ∼ Max		
Pre-T	41.00, 80.33	16.00~135.00	40.33, 77.00	16.00~135.00	-6.792	<0.001
T1	38.67, 68.67	18.33~117.00	38.33, 68.67	16.33~117.00	-12.043	<0.001
Pre-T & T1	50.67, 66.50	19.83~95.00	48.50, 66.00	18.83~95.00	-8.304	<0.001

**Table 4:** Exposure of PM10 in different groups of pre-trimester, first-trimester, and pre-trimester&first-trimester.

	PM10				Z value	P
	GDM		Non-GDM			
	M(P25, P75)	Min ∼ Max	M(P25, P75)	Min ∼ Max		
Pre-T	79.33, 134.33	35.67~212.33	78.00, 134.33	35.67~212.33	-5.199	<0.001
T1	78.00, 120.67	42.00~197.00	76.33, 123.00	35.67~200.67	-8.927	<0.001
Pre-T & T1	94.17, 119.00	45.67~165.67	87.50, 119.00	43.83~165.67	-5.753	<0.001

**Table 5:** Exposure of PM2.5 in different groups of per month.

	PM2.5				Z value	P
	GDM		Non-GDM			
	M(P25, P75)	Min ∼ Max	M(P25, P75)	Min ∼ Max		
1month of Pre-T	37.00, 71.00	14.00~150.00	37.00, 67.00	14.00~150.00	-5.788	<0.001
2month of Pre-T	37.00, 72.00	14.00~200.00	37.00, 72.00	14.00~200.00	-3.909	<0.001
3month of Pre-T	37.00, 77.00	14.00~150.00	37.00, 75.00	14.00~150.00	-3.466	0.001
1month of T1	37.00, 71.00	14.00~150.00	36.00, 66.00	14.00~150.00	-10.704	<0.001
2month of T1	36.00, 46.00	14.00~150.00	36.00, 65.00	14.00~150.00	-9.517	<0.001
3month of T1	34.00, 71.00	13.00~150.00	34.00, 66.00	13.00~150.00	-8.219	<0.001

**Table 6:** Exposure of PM10 in different groups of per month.

	PM10				Z value	P
	GDM		Non-GDM			
	M(P25, P75)	Min ∼ Max	M(P25, P75)	Min ∼ Max		
1month of Pre-T	72.00, 128.00	32.00~232.00	72.00, 128.00	32.00~232.00	-5.997	<0.001
2month of Pre-T	72.00, 135.00	32.00~303.00	72.00, 135.00	32.00~303.00	-3.387	0.001
3month of Pre-T	72.00, 132.00	32.00~232.00	72.00, 133.00	32.00~232.00	-3.119	0.002
1month of T1	73.00, 122.00	32.00~232.00	72.00, 122.00	32.00~232.00	-7.582	<0.001
2month of T1	73.00, 120.00	32.00~232.00	72.00, 120.00	32.00~232.00	-7.875	<0.001
3month of T1	71.00, 120.00	29.00~232.00	69.00, 120.00	29.00~232.00	-6.895	<0.001

### The correlation between PM2.5 exposure and GDM in different periods

**Figure 1:**
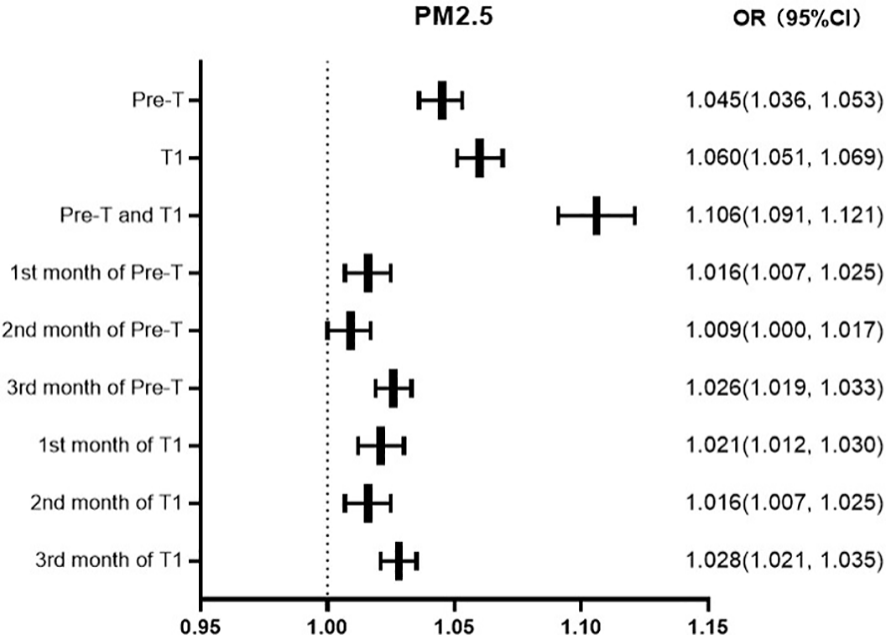
Forest map of PM2.5 exposure OR values at different periods.

GDM was defined as the dependent variable (0=no, 1=yes), and the factors of age, marital status, times of gravidity and parity, gestational hypertension, and anemia were defined as independent variables. Binary logistic regression analysis was used for analysis. The regression results show that, with the same other factors, the risk of GDM increases by 4.5%, 6.0%, and 10.6% for every 10ug/m3 increase in the average exposure value of PM2.5 in Pre-T, T1, Pre-T and T1, respectively, and 1.6%, 0.9%, and 2.6% respectively in the first, second, and third months of Pre-T and T1.1%, 1.6%, 2.8% respectively in the first, second, and third months of T1 (**Figure 1**).

### The correlation between PM10 exposure and GDM in different periods

**Figure 2:**
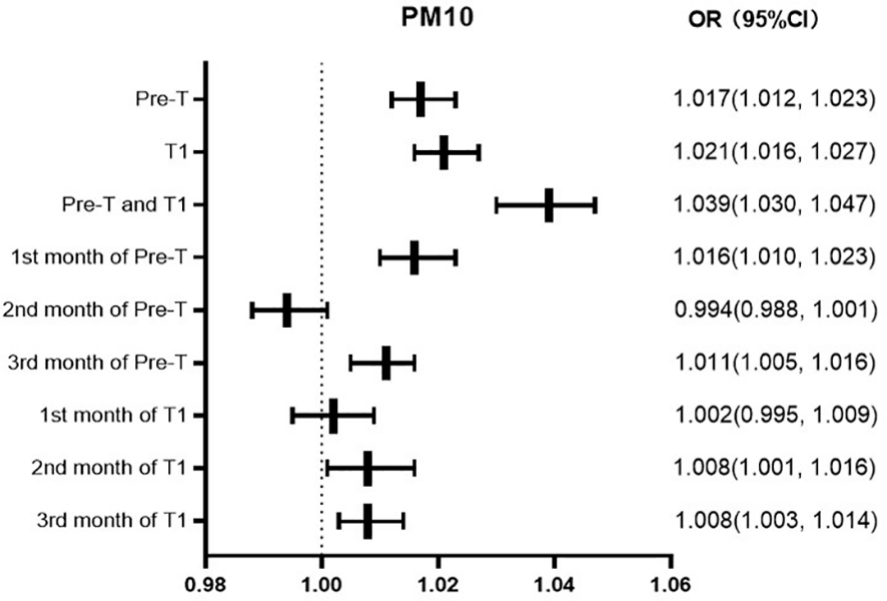
Forest map of PM10 exposure OR values at different periods.

The risk of GDM increases by 1.7%, 2.1%, and 3.9% for every 10ug/m3 increase in the average exposure value of PM10 in Pre-T, T1, Pre-T and T1, respectively. The risk of GDM increases by 1.6% and 1.1% for every 10ug/m3 increase in the average PM2.5 exposure value in the first and third months of Pre-T, respectively. While there was not statistically significant in the second month of Pre-T. The risk increased by 2.0%, 0.8%, and 0.8% in the first, second, and third months of T1, respectively (**Figure 2**).

## Discussion

From 2013 to 2018, the prevalence rate of diabetes in China increased from 10.9% to 12.4%, and the overall prevalence rate of adult diabetes and pre-diabetes has reached 50.5% (10). At the same time, the prevalence of GDM is also rising. The prevalence of GDM in China shows that the prevalence of GDM is 11.91% (3) The average incidence of GDM in Hebei from 2014 to 2021 was 7.64% (11). In this study, the prevalence of GDM increased over the course of the study from 10.2% in 2019 to 14.9% in 2021. How to prevent the occurrence of GDM and reduce the disease burden of diabetes is a serious public health challenge at present.

GDM refers to the abnormal glucose metabolism of pregnant women firstly found in 24-28 weeks of pregnancy, which is one of the most common complications of pregnancy (12). GDM will not only increase the incidence rate of adverse perinatal maternal and infant outcomes, but also increase the long-term risk of obesity, type 2 diabetes and cardiovascular disease in the offspring (2, 11).

The etiology of GDM was complicated and this hyperglycemia is the result of impaired glucose tolerance due to pancreatic β-cell dysfunction on a background of chronic insulin resistance (13). In recent years, the potential risks of air pollution to human health have become a research hotspot. Air pollution is one of the most serious environmental problems in the world. Air pollution is one of the most serious environmental problems in the world, and toxicological studies have shown that air pollutants can cause widespread damage to the respiratory system, cardiovascular system, immune system, and endocrine system (14–19). Moreover, systemic inflammation, oxidative stress, and endothelial dysfunction have been proposed (6, 7). Systemic inflammation and oxidative stress induced by air pollution can lead to insulin resistance, which is the underlying mechanism of gestational diabetes mellitus (GDM) in pregnant women (8, 20, 21).

Due to differences in geographical environment, industrial structure, and other aspects, the air pollution situation in various cities in Hebei Province is polarized. Data shows that Handan City, Shijiazhuang City, and Xingtai City have consistently ranked among the top three cities in terms of PM2.5 and PM10 concentrations, while Zhangjiakou, Chengde, and Qinhuangdao have concentrations of PM2.5 and PM10 that meet or approach the national secondary standard for ambient air quality. Therefore, pregnant women in various regions of Hebei Province face an external environment with significant differences in PM2.5 and PM10 concentrations, which is helpful for stratified analysis of the impact of PM2.5 and PM10 exposure on GDM during pregnancy or while trying to get pregnant.

Recent studies (22–24) have shown a close correlation between air pollution exposure and the occurrence of GDM; However, the research conclusions of the association between air pollutant exposure and the incidence of diabetes in pregnancy are inconsistent, and the window period of pollutant exposure is also unclear. Research (25–30) shows that air pollution can induce oxidative stress, inflammatory response, and lipid metabolism dysregulation of adipokines and imbalance of gut microbiota, ultimately leading to abnormal glucose metabolism and insulin resistance which might induce GDM. Our study found that exposure to PM2.5 and PM10 during the period of preparation and first-trimester of pregnancy was associated with GDM. This is consistent with previous research results (31–34). Systemic inflammation, oxidative stress dysregulation of adipokines, and imbalance of gut microbiota induced by air pollution can lead to insulin resistance, which is the underlying mechanism of gestational diabetes mellitus (GDM) in pregnant women (8).

Previous studies (34, 35) on air pollution and GDM have mostly focused on the first and second trimester of pregnancy. And research shows that pre pregnancy may also be an important exposure window period for air pollution exposure to affect GDM (36). Multivariate logistic regression analysis showed that the risk of GDM increases by 4.5%, 6.0%, and 10.6% for every 10ug/m3 increase in the average exposure value of PM2.5 in preconception, first trimester, preconception and first trimester. And this is consistent with previous research results. Moreover, we have conducted a detailed analysis of the impact of each month on GDM occurrence, refining the exposure period to more accurately determine the critical exposure window. Particulate pollution varies in size, shape, and composition. Excepting PM2.5 and PM10, particulate pollution can be made up of a variety of components including acids, inorganic compounds, organic chemicals, soot, metals, soil or dust particles, and biological materials. These components may also be crucial to accurately assess their health effects, especially in vulnerable populations such as pregnant women. In future work, we will also further investigate the impact of other pollutants on the pregnancy outcomes of pregnant women.

## Conclusions

GDM remains to be the most common metabolic disturbance during pregnancy, which has significant short and long-term complications for both the mother and the offspring. Exposure to high concentrations of PM2.5 and PM10 can increase the risk of GDM. Women in high-risk areas should pay attention to prenatal protection, especially during the preparation and early stages of pregnancy. We can develop the habit of checking the air quality index report every day to keep abreast of the level of particulate matter pollution in real time. If the air quality is poor, we can try not to go out or take protective measures when going out. In addition, we can also use air purifiers and other methods to reduce the inhalation of polluted air.

Our study had some limitations. This study is epidemiological and mechanism about the association between maternal exposure to air pollution and GDM should be deeply explored. And moreover, due to limitations of clinical data, the lack of clinical data on pregnant women’s height, weight, and weight gain during pregnancy can lead to analyzed biases. But this study has a large amount of data and high reliability of the experimental results.

## Data Availability

The raw data supporting the conclusions of this article will be made available by the authors, without undue reservation.
